# Advancing theories, models and measurement for an interprofessional approach to shared decision making in primary care: a study protocol

**DOI:** 10.1186/1472-6963-8-2

**Published:** 2008-01-03

**Authors:** France Légaré, Dawn Stacey, Ian D Graham, Glyn Elwyn, Pierre Pluye, Marie-Pierre Gagnon, Dominick Frosch, Margaret B Harrison, Jennifer Kryworuchko, Sophie Pouliot, Sophie Desroches

**Affiliations:** 1 Research Center of the Centre Hospitalier Universitaire de Québec, Québec, Canada; 2 Department of Family Medicine, Université Laval, Québec, Canada; 3 Faculty of Health Sciences, School of Nursing, University of Ottawa, Ottawa, Canada; 4 Vice-President of Knowledge Translation at Canadian Institute of Health Research; 5 Department of Primary Care and Public Health, School of Medicine, Cardiff University, Cardiff, UK; 6 Department of Family Medicine, McGill University, Montréal, Canada; 7 Department of Medicine, University of California, Los Angeles, Los Angeles, California, USA; 8 School of Nursing, Queen's University, Kingston, Canada

## Abstract

**Background:**

Shared decision-making (SDM) is defined as a process by which a healthcare choice is made by practitioners together with the patient. Although many diagnostic and therapeutic processes in primary care integrate more than one type of health professional, most SDM conceptual models and theories appear to be limited to the patient-physician dyad. The objectives of this study are to develop a conceptual model and propose a set of measurement tools for enhancing an interprofessional approach to SDM in primary healthcare.

**Methods/Design:**

An inventory of SDM conceptual models, theories and measurement tools will be created. Models will be critically assessed and compared according to their strengths, limitations, acknowledgement of interprofessional roles in the process of SDM and relevance to primary care. Based on the theory analysis, a conceptual model and a set of measurements tools that could be used to enhance an interprofessional approach to SDM in primary healthcare will be proposed and pilot-tested with key stakeholders and primary healthcare teams.

**Discussion:**

This study protocol is informative for researchers and clinicians interested in designing and/or conducting future studies and educating health professionals to improve how primary healthcare teams foster active participation of patients in making health decisions using a more coordinated approach.

## Background

With the increased emphasis on engagement of patients as partners in their care, there is a need to determine effective ways to involve them in the process by which healthcare decisions are made [1]. Effective clinical decisions are defined as the best course of action given the current scientific evidence, healthcare resources, clinical circumstances, and patient preferences [2]. It is expected that the need for patient guidance will only increase as clinical options multiply and health-related decision become more challenging given the need to weigh benefits and harms across options. Consequently, the processes by which patients are engaged to share their preferences and become involved in primary healthcare decisions are changing [3].

At the same time, primary healthcare services in Canada have been expanding to include other health professions, in addition to physicians [4]. Starfield (1998) defines primary care as "a health service system that provides entry into the system for all new needs and problems, provides person-focused (not disease-oriented) care over time, provides care for all but very uncommon or unusual conditions, and coordinates or integrates care provided elsewhere or by others [5]." While primary care in Canada refers to "the diagnosis, treatment and management of health problems with services delivered largely by physicians," it is also recognised as encompassing the broader determinants of health such as sickness prevention and health promotion activities that are provided by physicians and others in a team-based environment [6]. In 2002, the Commission on the Future of Health Care in Canada concluded that primary healthcare and prevention needed to be strengthened. The key components of the newly proposed primary healthcare services in Canada are: (a) team approach to service delivery; (b) roster of patients; (c) twenty-four hour access, seven days a week; (d) mixed funding formulas for services and programs; and (e) increased emphasis on health promotion and prevention [7]. Therefore, an interprofessional approach for the delivery of primary healthcare services in Canada is unavoidable. The following study protocol was developed to enlarge the scope of shared decision making to an interprofessional perspective and be responsive to the decision making needs of Canadians within primary health care services.

SDM is defined as a process by which a healthcare choice is made by practitioners together with the patient and is said to be the crux of patient-centered care [8,9]. A recent systematic review of SDM as a concept identified 161 conceptual definitions and summarized the key elements in one integrative model of SDM in medical encounters (Table 1) [10]. However, none of the identified definitions or the proposed models included an interprofessional perspective. The authors of this systematic review concluded that it was "equally important to study communication and decision-making in relatively mundane contexts such as primary healthcare," suggesting that SDM in primary health care contexts had not been satisfactorily addressed [10].

**Table 1:** Summary of key elements within SDM definitions based on the work by Makoul and Clayman (2005) [10]

**Essential elements**	**Ideal elements**	**General qualities**
• Definition/explanation of problem • Presentation of options • Discussion of pros/cons (benefits/risks/costs) • Explication of patient values/preferences • Discussion of patient ability/self-efficacy • Presentation of doctor knowledge/recommendations • Checking/clarifying understanding • Making or explicitly deferring decision • Arranging follow-up	• Presentation of unbiased information • Definition of roles (desire for involvement) • Presentation of evidence • Reaching mutual agreement	• Deliberation/negotiation Flexibility/individualized approach • Information exchange • Involves at least two people • Middle ground • Mutual respect • Partnership • Patient education • Patient participation • Process/stages

Globally, two main types of models of SDM have been proposed: transactional (e.g., focused on patient-practitioner roles in SDM) [11,12] and descriptive (e.g., focused on structuring decision support to address expectations, values, and other influential determinants of decision making) [13,14]. However, these models mostly address issues related to how the dyad (e.g., almost exclusively patient and physician) operationalized SDM. Indeed, most conceptual models and theories of SDM are essentially focused on the process by which decisions are made within a specific clinical encounter that is when one health practitioner meets with a specific patient. Nonetheless, these models form the basis for an inventory of the various theories and conceptual models of SDM. Furthermore, this inventory can provide a rich source of theoretical models to be critically reviewed using theory analysis (e.g, characteristics, strengths, weaknesses).

Health services-related policy documents in Canada clearly indicate the need for an interprofessional approach to providing patient-centered care [15-19]. An interprofessional approach is a process by which professionals collaborate to provide integrated and cohesive patient care [20,21]. Professionals include any healthcare workers involved in patient care across the spectrum from prevention to treatment and/or rehabilitation. Based on a review of conceptual frameworks and case studies of interprofessional collaboration, key elements influencing successful implementation of interprofessional practice have been identified at the practitioner level (e.g., partnerships, effective communication, power), organisational level (e.g., leadership, training and development, processes, division of labour), and socio-political level (e.g., leadership, funding, pre-licensure uni-professional educational environments) [20,22]. A systematic review of the evidence revealed that interprofessional collaborative practice significantly improves patient (e.g., reduced mortality, enhanced healthy function) and process outcomes [23]. However, less is known about the influence of pre-licensure interprofessional education on practice and patient outcomes [23]. Therefore, this provides evidence of the growing importance underlying interprofessional approaches for improving patient care. As well, interprofessionalism can be used as a lens to explore SDM within this broader perspective of the healthcare team approach to patient-centered care [24].

According to the Interprofessional Education for Collaborative Patient-Centered Practice Model [20,21], interprofessional education learning environments need to be considered concurrently with interprofessional collaborative practice, including linkages between these worlds. Furthermore, this model proposes that professionals need to reconcile their differences and synergistically influence patient care through continuous interaction, sharing of their knowledge, and optimizing patients' participation. Interprofessional collaborations build on the strengths of each profession's approach to care delivery such that professionals function within their full scope of practice without intentional duplication of services [25]. However, a review of the relevant conceptual models and collaborative practice as a core concept underlying interprofessional practice highlights the need to further develop patient participation within interprofessional collaboration [20].

Indeed, in a recent review, we identified 31 publications of 28 unique studies in 15 countries that reported on health professionals perceived barriers and facilitators to implementing SDM in clinical practice [26]. Overall, the vast majority of participants (n = 2784) were physicians (89%) suggesting that there was a lack of interprofessional perspective to SDM. Interestingly, the most often reported barrier was time constraints (18/28) thus reinforcing the need to foster a more coordinated interprofessional effort for implementing SDM in clinical practice.

As well, we recently completed a study funded by the Ontario Ministry of Health and Long-term Care which aimed at improving the quality and cost-effectiveness of decision support provided by primary health professionals in Family Health Networks in Ontario. Although, interventions were targeted at the patients, nurses, and physicians individually, none of the interventions used interprofessional education or strategies to enhance collaborative practice. Findings suggested that providing decision support in a collaborative and coordinated manner to patients was not natural for family physicians and nurses [27]. Therefore, an interprofessional approach to SDM has the potential to improve the quality of patient decision support provided within primary care. This would involve professionals: (a) sharing the common goal of achieving quality health decisions that are informed and based on patients' values; (b) having a sense of trust among the different professionals participating in the process by which the decision is made; (c) being governed by leaders that value SDM; and (d) having organisational structures to facilitate implementing SDM within the processes of care.

Overall, the context and current policies in Canada reinforce the role of primary healthcare and the need for interprofessional teams to address new challenges in healthcare including: (1) explosion of health information [28]; (2) expanding role and participation of patients in clinical decision-making [29]; (3) increasing burden of chronic diseases[7] and multimorbidity in patients seen in primary healthcare clinical settings [30]; and (4) shortage and constraints in the health labour force [31-33]. However, there are important unresolved issues related to appropriate conceptual models, theories and measurement tools for conducting applied health services research, as well as for enhancing best practices and training healthcare providers to use an interprofessional approach to SDM in primary healthcare. These processes of decision-making are changing with increasing emphasis on the patient's role and on interprofessional perspective. Therefore, an interprofessional approach to SDM is the process by which patients are supported to become involved in decision-making, have their decisional needs met, and reach healthcare choices that are agreed upon by them and their practitioners. To date, known SDM conceptual models and theories are limited to the patient-physician dyad [11-14] and most SDM initiatives have targeted individual professional groups [34-36] and/or the evaluation of patient decision aids [37]. In addition, as healthcare systems are increasingly aiming at patient-centered primary care [38], a philosophy of care that aims at the best possible integration of the patient's perspective [39,40], fostering the engagement of patients in the process of SDM with a broader range of health professionals and disciplines will be fundamental [41]. Finally, the existing literature points to a lack of guidance in how the existing conceptual models, theories, and measurement tools in SDM and interprofessionalism relate to enhancing current practice, applied health services research, and training activities to support an interprofessional approach to SDM in primary healthcare.

Therefore, the main objective of this project is to develop and validate with key stakeholders a conceptual model and propose a set of measurement tools for enhancing an interprofessional approach to SDM in primary healthcare practices, education, and applied health services research. The specific objectives that will be addressed are to:

1) Perform a theory analysis of the existing conceptual models and theories in SDM to ascertain their characteristics, strengths, and limitations, including their acknowledgement of interprofessional roles in the process of SDM, and the extent to which they have been tested in applied health services research and educational activities in primary healthcare;

2) Assess the validity and reliability of identified measurement tools that would be relevant to an interprofessional approach to SDM in primary healthcare practices, education, and applied health services research;

3) Achieve consensus among research team members for a new conceptual model and a set of measurement tools for an interprofessional approach to SDM in primary healthcare practices, education, and applied health services research;

4) Validate and identify the perceived barriers and facilitators to the implementation of the proposed conceptual model and set of measurement tools for an interprofessional approach to SDM in primary healthcare practices, education, and applied health services research with representatives from clinical practice, research, education and policy environments.

## Methods/Design

Guided by the framework for developing health policy recommendations [42,43] and based on the above-mentioned specific objectives, the present research project will address four sets of questions that are described with their proposed methods as follows [42].

### Set #1

How do the existing conceptual models and theories in SDM inform an interprofessional approach to SDM in primary health care? What are their characteristics, strengths, and limitations? To what extent have they been tested in applied health services research and educational activities?

Synthesis, derivation, and analysis are the three basic approaches to theory building [44]. Given the existing literature on the topic, access to the expertise and unique resources within the research team, and our personal judgment about which approach would be the most productive for the SDM community, this project will be guided by a theory analysis approach to theory building [44]. Theory analysis is a systematic examination of a theory or theories, and consists of an essential component of theory development [44].

Based on *a priori *selection criteria, an inventory will be created of SDM conceptual models, theories, and measurement tools. The initial inventory will be based on those known to team members as well as identified in published reviews. About 170 experts in the field will be contacted through an SDM electronic mailing list to update this inventory. Second, for each of the identified conceptual models, two reviewers will independently extract data using a standardized form, which includes key data necessary for theory analysis [44,45] and is based on the data collection checklist developed for a theory analysis of knowledge translation models/theories relevant to health care organisation and professional behaviour. Pilot testing of the standardized form will be conducted with team members and results discussed in a conference call to finalize the form. Pairs of reviewers will independently extract information using the standardized tool and compare findings with disagreements resolved through consensus or appeal to the co-principal investigators (FL, DS). Findings will be entered into a matrix to facilitate comparison of how each conceptual model performs on each item of interest:

• Identifying the origins of the conceptual model or theory: Who developed it? Where are they from (institution, discipline)? What prompted the originator to develop it? Is it inductive or deductive in form? Is there evidence to support or refute the nature of this conceptual model/theory? Year that it was developed/published? Dates for any subsequent revisions or modifications?

• Examining the meaning of the conceptual model/theory: What are the concepts? How are the concepts defined? What is the relationship between concepts (propositions)?

• Analyzing the logical consistency (logical structure of the concepts and statements) of the conceptual model/theory: Are there any logical fallacies in the structure of the conceptual model/theory? Is there a diagram or is it possible to draw a diagram of the key concepts and their relationships?

• Defining the degree of parsimony (how simply and briefly a conceptual model/theory can be stated in its explanation of the phenomenon in question) and generalizability of the conceptual model/theory: Can generalizations be made from it?

• Determining the testability of the conceptual model/theory: A conceptual model/theory that can not generate hypotheses to be subjected to empirical testing through research is defined as not testable: can the conceptual model/theory be supported with empirical data?

• Ascertaining the degree of adequacy and usefulness of the conceptual model or theory for exploring an interprofessional approach to SDM in clinical practice in primary health care or educational setting.

### Set #2

What are the validity and reliability of existing measurement tools that would be relevant to an interprofessional approach to SDM?

For each measurement tool that was identified in our inventory (see previous section), two reviewers will independently extract data using a standardized form. This data collection checklist is based on guidelines for instrument development and evaluation by McDowell and Newell [46] and by Tremblay and collaborators [47]. Pilot testing of the tool will be conducted to ensure consistent use of the tool across data collectors and verify clarity of the items. The standardized tool will be finalized in a conference call with reviewers. Pairs of reviewers will independently extract information using the standardized tool and compare results with disagreements resolved through consensus or appeal the co-principal investigators (FL, DS). Findings will be entered into a matrix that facilitates comparison of how each measurement tool performs on each item of interest. An analysis of the identified measurement tools will be performed and include:

• Identifying the purpose of the measurement tool: Is the purpose of the measurement tool fully explained? Where are they from (institution, discipline)? What prompted the originator to develop it? Is it appropriate for the intended use? Was the measurement tool developed and tested on the types of person to whom it will be applied?

• Examining the scope of the measurement tool: Is it broad enough for the application, asking neither too many nor too few questions? Is it relevant to interprofessional approach to SDM in primary health care practice or research?

• Analyzing the conceptual underpinnings of the measurement tool: What is the conceptual models/theory underlining the measurement tool? Is it well established? Does it correspond to a broader body of knowledge?

• Defining the degree to which the measurement tool is easy to administer: Does it appear that it is feasible to use this measurement tool? Is it too long? Is professional expertise required? Does it look as if this is acceptable for the target subjects/can it be self administered? What were the response rates that were achieved? Is there a cost involved to use the measurement tool? Is there clear instruction in the user's manual?

• Determining how clear the instructions are for scoring: Is it clear how the method is scored? Is the numerical quality of the scores suited to the type of statistical analyses planned? If the method uses an overall score, how is this to be interpreted?

• Ascertaining the degree of change that can be detected: Does the measurement tool detect qualitative change only or does it provide quantitative data?

• Ascertaining the available evidence for reliability and validity: How many different forms of quality testing have been carried out? How many other measurement tools has it been compared with? How many different users have tested the method and did they obtain similar results? How do these compare to the quality of other scales?

### Set #3

For an interprofessional approach to SDM in primary healthcare practices, education, and applied health services research, which new conceptual model and set of measurement tools can achieve consensus by the team members?

This research activity will involve developing, by consensus of the team members, a new conceptual model and a set of measurement tools for enhancing an interprofessional approach to SDM in primary healthcare practices, education, and applied health services research. Indeed, "a theory must be sufficiently precise in its representation for scientists to agree on the predictions that can be made from it [44]." Consensus building is defined as a process of seeking unanimous agreement [48]. Hence, the methods used will be based on consensus building methods and will include: (1) convening, (2) clarifying responsibilities, (3) deliberating, (4) deciding, and (5) implementing agreement.

The two co-principal investigators (FL, DS) will use results from the activities described in sections 'Set #1' and 'Set #2' to prepare a draft of a conceptual model and a set of measurement tools for enhancing an interprofessional approach to SDM in primary healthcare. The draft model set of measurement tools and the detailed results from these research activities will be formatted in a document that will be sent to team members in preparation for a face-to-face team meeting. This meeting will be structured around a specific goal: building on the existing conceptual models of SDM, to develop a conceptual model for an interprofessional approach to SDM in primary care. This two-day meeting will be under the responsibility of the two co-principal investigators (FL, DS) but will be lead by a facilitator who will be hired from a private consulting firm. The agenda will be based on consensus building methods [48] and will include the following steps:

• Clarification: Clarify the roles of the facilitator, team leaders (two co-principal investigators) and team members.

• Agenda and rules: Get agreement that the conceptual model to be developed will be inline with the items included in our critical appraisal tool and will need to meet the pre-determined criteria that are included in this appraisal tool (e.g., clear definition of concepts, logical, parsimonious, and testable) and specify a timeline (two days).

• Deliberation: Review the draft of a conceptual model and set of measurement tools for enhancing an interprofessional approach to SDM in primary healthcare, and pursue deliberations over the draft to seek suggestions for clarification and/or improvements to be made on the proposed conceptual model.

• Decision: Achieve agreement on the final version of the conceptual model and set of measurement tools for an interprofessional approach to SDM in primary healthcare.

• Implementation of agreement: Seek validation of key stakeholders (see research activity in the next section).

### Set #4

What do key stakeholders from the clinical practice, research, educational and policy environments think of our proposed conceptual model for an interprofessional approach to SDM in primary care? What are their opinions and views on it? What changes do they propose? What are the barriers and facilitators they perceive to its implementation?

A series of individual interviews and focus groups will be conducted with key stakeholders to explore face and content validity of the proposed conceptual model, suggestions for improvement and potential factors influencing implementation of the model. First, participants will be purposefully selected through the network contact method to fit within one of four distinct groups [49]:

• Key stakeholder organisations in Canada representing the professional, educational, policy environments, and the health consumers' perspective;

• A team of primary care clinicians who were involved in the past year in a study funded by the Ontario Ministry of Health and Long-term Care to improve the quality and cost-effectiveness of decision support provided by primary health professionals in Family Health Networks in Ontario;

• A team of primary care clinicians who are currently involved in a study funded by Health Canada to assess an interprofessional approach for training healthcare professionals in primary care;

• A team of primary care clinicians who never were involved in any study or implementation project regarding SDM nor interprofessional approaches (the naïve group).

Second, a document that includes the relevance and main objective of this study as well as the proposed conceptual model for enhancing an interprofessional approach to SDM in primary healthcare will be prepared and sent to participants before the interviews or focus groups. Third, with representatives of the key stakeholder organizations, seven to 10 individual telephone interviews will be conducted by the research coordinator. For example, the Canadian Nursing Association, College of Family Physicians of Canada, Health Canada, British Columbia Ministry of Health are among the organisations that have agreed to participate in this project.

With teams of primary care clinicians, three focus groups of five to eight participants will be conducted by one of the co-principal investigators (FL, DS) with the help of the research coordinator (SP) [50]. An interview guide will be used to facilitate a semi-structured discussion with the participants regarding the proposed conceptual model and a set of measurement tools for enhancing an interprofessional approach to SDM in primary healthcare. Questions in the interview guide are grounded in theory analysis [44], the Ottawa Model of Research Use (OMRU) [51,52] and a guide for health policy analysis [53]. According to the OMRU, the four phases for implementing innovations in practice are: (1) assessing the barriers and facilitators at the level of the innovation, potential adopters, and practice environment; (2) designing and implementing interventions based on the known barriers and facilitators; (3) measuring adoption of the innovation; and (4) evaluating patient, practitioner, and system outcomes. Examples of issues included within questions on the interview guide include: clarity of the core concepts, linkages between concepts, proposed changes, who they perceived would have a favourable/negative attitude regarding this model, and barriers and facilitators to implementing this model in clinical practice. All individual interviews and focus groups will be tape-recorded and transcribed verbatim. We will also collect information on the demographic characteristics of the participants and additional information on their organisations.

Analysis of the transcripts will occur concurrently with data collection in the focus groups and individual interviews using a constant comparative method. Constant comparative method is defined as categorizing units of data through a process of comparing new units with previously identified units to develop or saturate a category [54]. NVivo software for qualitative analyses will be used to support data organisation and analysis. The aim is to highlight needed changes to the conceptual model proposed by the research team and anticipated barriers and facilitators for implementing an interprofessional approach to SDM in primary care. With the aim of verifying credibility of the findings, a summary of the interpretations of the interviews and focus groups with suggested changes to the proposed conceptual model will be sent to each participant (member checking) [50]. They will be invited to make additional comments or corrections. Subsequently, suggested changes with proposed actions to be taken will be circulated and discussed in a conference call with the research team. A final version of the conceptual model and its set of measurement tools will be produced.

#### Ethical considerations

All data collected for this project will be obtained from publicly available materials for specific objectives (1) and (2). Participants for the individual interviews and focus groups will be asked to complete consent forms. Ethics approval for the project was received from the Research Ethics Board of the Centre Hospitalier Universitaire de Québec (approved May 17^th ^2007; ethics number 2007-2008-02).

## Discussion

The focus of this research will be to enlarge the scope of SDM to an interprofessional perspective to respond to the need for patient-centred approaches to involving patients in making decisions regarding primary healthcare services. It will use a novel approach to explore the relevance of the existing conceptual models, theories, and measurement tools of SDM for an interprofessional approach that fosters the active participation of patients to decisions in primary care. With the contribution of collaborators, the research team will then build upon these theoretical models to propose a conceptual model and a set of measurement tools for an interprofessional approach to SDM in primary healthcare. This research will add to scholarly knowledge regarding how SDM is conceptualized and measured but most importantly, it will be the starting point for addressing whether and how changing understanding of conceptual frameworks has the desired impact on health systems and organisations in relationship with other known barriers to SDM [26]. The study results and the conceptual model and set of measurement tools will be disseminated through publications in peer-reviewed journals and at conferences (both scientific and professional) having themes related to SDM, interprofessionalism and on the Best Practices in SDM website at Université Laval and on the Ottawa Health Decision Center website.

## Competing interests

The author(s) declare that they have no competing interests.

## Authors' contributions

FL, DS, IG, GE, PP, MPG, DF and MBH conceived the study, sought funding and ethical approval. FL, DS and SP are responsible for the management of the trial. JK, SP and SD are responsible for the data collection. All authors read, and approved the final manuscript. FL and DS are its guarantors.

## Pre-publication history

The pre-publication history for this paper can be accessed here:



## References

[B1] The Change Foundation in partnership with IBM Business Consulting Consumers and Canadian Health Care Trending Analysis 2004. Report Number February Available at. http://www.changefoundation.com.

[B2] Sackett DL (1997). Evidence-based medicine. Semin Perinatol.

[B3] Coulter A (1999). Paternalism or partnership? Patients have grown up-and there's no going back. BMJ.

[B4] D'Amour D (2002). The future of collaboration between nurses and family physicians. Infirm Que.

[B5] Starfield B (1998). Primary Care Balancing Health Needs, Services, and Technology.

[B6] Fooks C (2003). Implementing Primary Care Reform in Canada: Barriers and Facilitators.

[B7] Health Council of Canada. 2006 Annual Report: The Road to Quality. http://healthcouncilcanada.ca/.

[B8] Towle A, Godolphin W (1999). Framework for teaching and learning informed shared decision making. BMJ.

[B9] Weston WW (2001). Informed and shared decision-making: the crux of patient centred care. CMAJ.

[B10] Makoul G, Clayman ML (2005). An integrative model of shared decision making in medical encounters. Patient Educ Couns.

[B11] Charles C, Gafni A, Whelan T (1997). Shared decision-making in the medical encounter: what does it mean? (or it takes at least two to tango). Soc Sci Med.

[B12] Whitney SN (2003). A new model of medical decisions: exploring the limits of shared decision making. Med Decis Making.

[B13] O'Connor AM, Tugwell P, Wells GA, Elmslie T, Jolly E, Hollingworth G, McPherson R, Bunn H, Graham I, Drake E (1998). A decision aid for women considering hormone therapy after menopause: decision support framework and evaluation. Patient Educ Couns.

[B14] Rothert ML, Talarczyk GJ (1987). Patient Compliance and the Decision-Making Process of Clinicians and Patients. The Journal of Compliance in Health Care.

[B15] Interprofessional Education for Collaborative Patient-Centred Practice Research Synthesis Paper. http://www.hc-sc.gc.ca/hcs-sss/hhr-rhs/strateg/interprof/synth_e.html.

[B16] Curran VR, Deacon DR, Fleet L (2005). Academic administrators' attitudes towards interprofessional education in Canadian schools of health professional education. J Interprof Care.

[B17] Villeneuve M, MacDonald J (2006). Toward 2020: visions for nursing setting the stage for the future. Can Nurse.

[B18] Primary Health Care Transition Fund. http://www.hc-sc.gc.ca/hcs-sss/prim/phctf-fassp/index_e.html.

[B19] Maxted JM (2005). Primary health care transition fund update. Can Fam Physician.

[B20] D'Amour D, Ferrada-Videla M, San Martin Rodriguez L, Beaulieu MD (2005). The conceptual basis for interprofessional collaboration: core concepts and theoretical frameworks. J Interprof Care.

[B21] D'Amour D, Oandasan I (2005). Interprofessionality as the field of interprofessional practice and interprofessional education: an emerging concept. J Interprof Care.

[B22] Irvine R, Kerridge I, McPhee J, Freeman S (2002). Interprofessionalism and ethics: consensus or clash of cultures?. J Interprof Care.

[B23] Zwarenstein M, Reeves S, Perrier L (2005). Effectiveness of pre-licensure interprofessional education and post-licensure collaborative interventions. J Interprof Care.

[B24] Prada G, Swettenham J, Stonebridge C, Schaafsma M-E, Grimes K, Thompson V Interdisciplinary Primary Health Care: Finding the answers – A case study report. The Enhancing Interdisciplinary Collaboration in Primary health care (EICP).

[B25] Way D, Jones L, Baskerville B, Busing N (2001). Primary health care services provided by nurse practitioners and family physicians in shared practice. CMAJ.

[B26] Gravel K, Legare F, Graham ID (2006). Barriers and facilitators to implementing shared decision-making in clinical practice: A systematic review of health professionals' perceptions. Implement Sci.

[B27] Jacobsen MJ, O'Connor A, Hogg B, Stacey D, Légaré F, Graves E, Bayne S, Saarimaki A, Drake E (2007). Improving the quality of decision support provided by primary care teams in Ottawa. 4th International Shared Decision Making Conference; Freiburg, Germany.

[B28] Woolf SH, Chan EC, Harris R, Sheridan SL, Braddock CH, Kaplan RM, Krist A, O'Connor AM, Tunis S (2005). Promoting informed choice: transforming health care to dispense knowledge for decision making. Ann Intern Med.

[B29] Kiesler DJ, Auerbach SM (2006). Optimal matches of patient preferences for information, decision-making and interpersonal behavior: Evidence, models and interventions. Patient Educ Couns.

[B30] Fortin M, Bravo G, Hudon C, Vanasse A, Lapointe L (2005). Prevalence of multimorbidity among adults seen in family practice. Ann Fam Med.

[B31] Canadian Policy Research Networks Inc Health Human Resource Planning in Canada. Physician and Nursing Work Force Issues. Report Number Commission on the Future of Health Care in Canada Ottawa October 2002.

[B32] Martin S (2002). More hours, more tired, more to do: results from the CMA's 2002 Physician Resource Questionnaire. CMAJ.

[B33] Watson DE, Katz A, Reid RJ, Bogdanovic B, Roos N, Heppner P (2004). Family physician workloads and access to care in Winnipeg: 1991 to 2001. CMAJ.

[B34] Stacey D, Graham I, O'Connor AM, Pomey M (2005). Barriers and facilitators influencing call center nurses' decision support for callers facing values-sensitive decisions: a mixed methods study. Worldviews on Evidence-Based Nursing.

[B35] Légaré F, O'Connor A, Graham I, Saucier D, Côté L, Cauchon M, Paré L (2006). Supporting patients facing difficult health care decisions. Use of the Ottawa Decision Support Framework. Can Fam Physician.

[B36] Elwyn G, Edwards A, Hood K, Robling M, Atwell C, Russell I, Wensing M, Grol R, The Study Steering G (2004). Achieving involvement: process outcomes from a cluster randomized trial of shared decision making skill development and use of risk communication aids in general practice. Fam Pract.

[B37] O'Connor AM, Stacey D, Entwistle V, Llewellyn-Thomas H, Rovner D, Holmes-Rovner M, Tait V, Tetroe J, Fiset V, Barry M, Jones J (2003). Decision aids for people facing health treatment or screening decisions. Cochrane Database Syst Rev.

[B38] Interprofessional education for collaborative patient-centered practice: An evolving Framework. http://www.hc-sc.gc.ca/hcs-sss/hhr-rhs/strateg/interprof/summ-somm_e.html.

[B39] Stewart M, Belle Brown J, Weston W, McWhinney IR, McMillan CL, Freeman TR (1995). Patient-Centered Medicine Transforming the Clinical Method.

[B40] Institute of Medicine Crossing the Quality Chasm: A New Health System for the 21st Century.

[B41] Harkness J (2005). Patient involvement: a vital principle for patient-centred health care. World Hosp Health Serv.

[B42] Dobrow M, Goel V, Lemieux-Charles L, Black N (2006). The impact of context on evidence utlization: A framework for expert groups developing health policy recommendations. Soc Sci Med.

[B43] Dobrow MJ, Goel V, Upshur RE (2004). Evidence-based health policy: context and utilisation. Soc Sci Med.

[B44] Walker L, Avant K (2005). Strategies for theory construction in nursing.

[B45] Fawcett J (1989). Conceptual Models and Theories. Analysis and Evaluation of Conceptual Models of Nursing.

[B46] McDowell I, Newell C (1987). Measuring Health A guide to rating scales and questionnaires.

[B47] Tremblay LE, Savard J, Casimiro L, Tremblay M (2004). Répertoire des outils d'évaluation en français pour la réadaptation.

[B48] Susskind L, McKearnan S, Thomas-Larmer J (1999). The Consensus Building Handbook.

[B49] Morse J, Denzin N, Lincoln Y (1994). Designing funded qualitative research. Handbook of qualitative research.

[B50] Krueger R (2006). Is it a Focus Group? Tips on How to Tell. J Wound Ostomy Continence Nurs.

[B51] Logan J, Graham ID (1998). Toward a Comprehensive Interdisciplinary Model of Health Care Research Use. Science Communication.

[B52] Graham K, Logan J (2004). Using the Ottawa Model of Research Use to implement a skin care program. J Nurs Care Qual.

[B53] O'Neill M, Gosselin P, Boyer M (1997). La santé politique Petit manuel d'analyse et d'intervention politique dans le domaine de la santé.

[B54] Creswell JW (1998). Qualitative inquiry and research design Choosing among five traditions.

